# Efeitos da infiltração intra-articular de aspirado de medula óssea no tratamento da osteoartrite de joelho: Estudo clínico comparando BMA versus corticosteroide e bloqueio genicular

**DOI:** 10.1055/s-0045-1809512

**Published:** 2025-06-14

**Authors:** Renata Clazzer, Dilamar Moreira Pinto, Mariana Valois de Aquino Krause, Tale Lucas Vieira Rolim, Ricardo Lyra de Oliveira, Diego Ariel de Lima

**Affiliations:** 1 Hospital Otavio de Freitas, Recife, PE, Brasil.; 2 Departamento de Ciências da Saúde, Universidade Federal Rural do Semi-Árido, Mossoró, RN, Brasil.

**Keywords:** injeções intra-articulares, manejo da dor, medula óssea, osteoartrite de joelho, bone marrow, injections, intra-articular, osteoarthritis, knee, pain management

## Abstract

**Objetivo** Avaliar a eficácia do aspirado de medula óssea autólogo (BMA) na redução da dor e melhora da funcionalidade em pacientes com osteoartrite (OA) de joelho, comparando com a infiltração intra-articular com corticoide e bloqueio genicular.

**Métodos** Um estudo clínico prospectivo, randomizado, controlado, simples-cego, comparativo e analítico foi realizado. Foram 50 pacientes com OS de joelho, divididos em dois grupos: um grupo de intervenção submetido ao tratamento com BMA e um grupo controle submetido à infiltração articular padrão com corticoide e bloqueio genicular. Os resultados foram avaliados usando o Índice de Osteoartrite das Universidades de Western Ontario e McMaster (WOMAC).

**Resultados** Após 6 meses, uma redução significativa da dor foi observada no grupo BMA em comparação ao grupo controle ( *p* = 0.030). Não foram encontradas diferenças significativas nos escores de rigidez e atividade física entre os grupos. O grupo de intervenção demonstrou melhorias significativas em todas as subcategorias do WOMAC avaliadas antes e após o tratamento.

**Conclusão** O tratamento com BMA pode reduzir significativamente a dor, possibilitando uma melhora funcional, sugerindo seu potencial como opção terapêutica viável no manejo da OA de joelho.

## Abstract (en)

**Objective** To assess the efficacy of autologous bone marrow aspirate (BMA) in reducing pain and improving functionality in patients with knee osteoarthritis (OA), in comparison with intraarticular corticosteroid injection and genicular nerve block.

**Methods** A prospective, randomized, controlled, single-blinded, comparative, and analytical clinical study was conducted. There were 50 patients with knee OA divided into two groups: an intervention group receiving BMA treatment and a control group undergoing standard corticosteroid articular infiltration and genicular block. Outcome measures were evaluated using the Western Ontario and McMaster universities osteoarthritis index (WOMAC).

**Results** After 6 months, significant pain reduction was noted in the BMA group compared with the control group ( *p* = 0.030). No significant differences were found in stiffness and physical activity scores between the groups. The intervention group demonstrated significant improvements in all assessed WOMAC subcategories pre- and posttreatment.

**Conclusions** Treatment with BMA can significantly reduce pain, potentially leading to improved knee functionality, suggesting its potential as a viable therapeutic option for managing knee OA.

## Introdução

A osteoartrite do joelho (OA) é uma doença degenerativa crônica que afeta predominantemente mulheres e provoca a deterioração progressiva da cartilagem articular. Esta condição leva a deformidades articulares e possíveis desequilíbrios musculares e ligamentares, em particular nas áreas sujeitas a maior carga, como evidenciado por características radiográficas típicas, incluindo esclerose óssea, cistos e osteófitos. 1 2 3

A OA do joelho influencia significativamente o desempenho físico e é considerada uma das dez principais causas de incapacidade no mundo. As abordagens terapêuticas conservadoras mais empregadas nessa doença incluem perda de peso, exercícios físicos, administração de anti-inflamatórios não esteroidais (AINEs), analgésicos, injeções intra-articulares de ácido hialurônico (AH) e glicocorticoides e bloqueios geniculares. 4

Recentemente, as injeções ortobiológicas surgiram como uma opção potencialmente segura e eficaz para o tratamento da OA do joelho, incluindo aspirado de medula óssea (BMA), células-tronco mesenquimais (MSCs) e plasma rico em plaquetas (PRP). 5 O uso do BMA como terapia celular inovadora se destaca por sua técnica simples, baixa morbidade e fornecimento de MSCs. Essas células têm a capacidade de promover o reparo do tecido articular e influenciar a expressão das citocinas interleucina (IL)-8 e -1β, além de servirem como fonte de peptídeos de sinalização intracelular, como o fator de crescimento derivado de plaquetas (PDGF), o fator transformador do crescimento β (TGF-β) e o fator de crescimento endotelial vascular (VEGF). 6 7 Assim, as injeções ortobiológicas podem ser uma excelente opção no tratamento da gonartrose.

Portanto, o objetivo deste estudo foi avaliar a eficácia do uso de BMA autólogo para redução da dor e melhora da funcionalidade em pacientes com OA de joelho em comparação à injeção intra-articular de corticosteroide e bloqueio do nervo genicular.

## Materiais e Métodos

Após a aprovação pelo Comitê de Ética em Pesquisa (CAAE: 1164923.6.0000.5200), foi realizado um estudo clínico prospectivo, randomizado, controlado, longitudinal, simples-cego (avaliadores), comparativo, descritivo e analítico. Este estudo envolveu pacientes com OA de joelho no serviço de ortopedia de nossa instituição. Os pacientes foram divididos em dois grupos por meio de randomização em blocos; a única especificação foi o número equivalente de pacientes em cada grupo. O grupo 1 (intervenção) recebeu tratamento com BMA e o grupo 2 (controle) foi submetido à infiltração articular de corticoides e bloqueio genicular, padrões na instituição. Todos os procedimentos foram realizados pelo mesmo cirurgião.

O número de participantes da amostra foi estipulado para garantir um intervalo de confiança (IC) de 95%, um poder de aproximadamente 80% e uma diferença entre grupos de 20%, ou seja, 25 pessoas no grupo intervenção e 25 no grupo controle, totalizando 50 indivíduos. 8

Os critérios de inclusão foram pacientes entre 30 e 90 anos, com de OA de graus II a IV pela escala de Kellgren e Lawrence, 9 ausência de outras doenças reumáticas inflamatórias, sem tratamento prévio com corticoides injetáveis ou orais nos últimos 12 meses e que assinaram o termo de consentimento livre e esclarecido.

Os critérios de exclusão foram aqueles com qualquer doença que impedisse o acompanhamento, perda de acompanhamento/contato com o paciente, uso de corticosteroides orais ou intravenosos durante o período de acompanhamento, hemoglobina menor que 11 g/dL, número de plaquetas inferior a 150.000/mm ^3^ , ou presença de qualquer distúrbio de coagulação.

### Grupo 1 (Intervenção): Tratamento com BMA

**Fig. 1:**
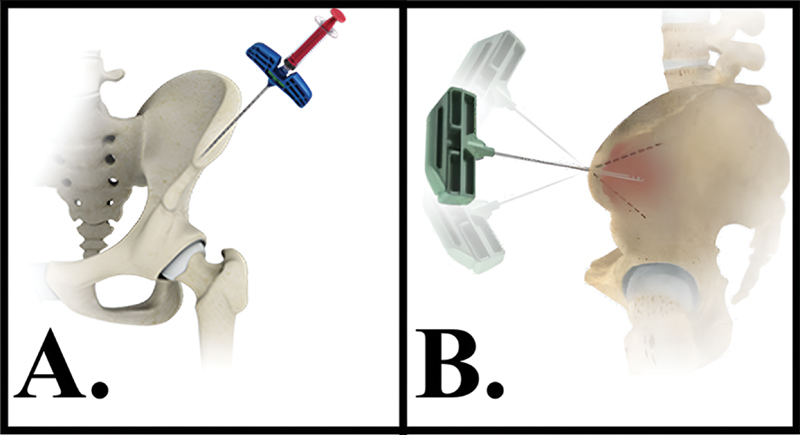
Aspirado de medula óssea (BMA). ( **A** ) Punção na crista ilíaca perto da espinha ilíaca anterossuperior, com uma inclinação de 40° de lateral para medial e 40° de superior para inferior. ( **B** ) A agulha pode ser retirada e reinserida com uma ligeira mudança na angulação para aspirar a medula óssea de outro sítio, o que pode melhorar a concentração de MSCs.

Sob orientação ultrassonográfica, aproximadamente 15 mL de BMA foram coletados da espinha ilíaca anterossuperior em seringas heparinizadas, usando uma técnica de baixo volume e múltiplos locais, 10 como mostrado nas Figs. 1 e 2 . A coleta de BMA foi realizada com o paciente sob sedação e anestesia local com lidocaína a 1% utilizando uma agulha/cânula de biópsia de medula óssea de 11 G.

**Fig. 2:**
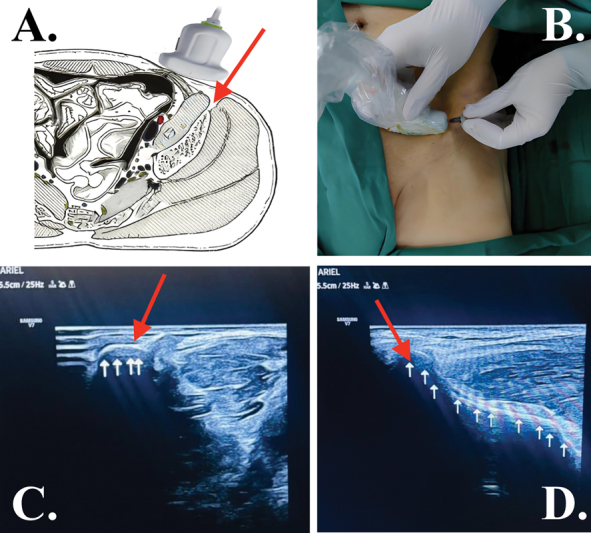
Aspirado de medula óssea (BMA). ( **A** ) Figura esquemática mostrando a sonda de ultrassom alinhada transversalmente ao osso ilíaco. ( **B** ) Imagem do paciente mostrando a sonda de ultrassom alinhada transversalmente ao osso ilíaco. ( **C** ) Imagem ultrassonográfica correspondente à posição da sonda alinhada transversalmente ao osso ilíaco. ( **D** ) Ao girar a sonda 90°, a imagem ultrassonográfica correspondente à posição da sonda alinhada longitudinalmente com o osso ilíaco. Seta vermelha, sítio de punção ilíaca; setas brancas, osso cortical ilíaco.

**Fig. 3:**
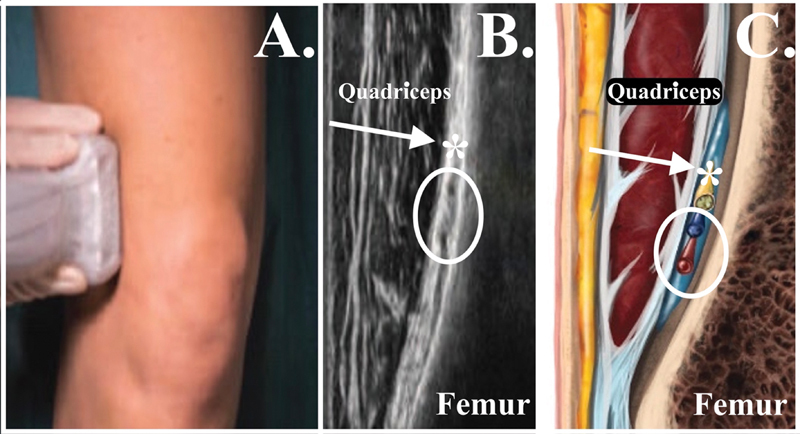
Pontos anatômicos para o bloqueio do nervo genicular superior lateral (LSGN). ( **A** ) Transdutor no plano coronal na região metafisária lateral distal do fêmur. ( **B** ) Imagem ultrassonográfica obtida com o transdutor no plano coronal na região metafisária lateral distal do fêmur. ( **C** ) Imagem esquemática no plano coronal na região metafisária lateral distal do fêmur. Seta branca e *, nervo genicular superior lateral; círculo branco, veia e artéria genicular superior lateral.

**Fig. 4:**
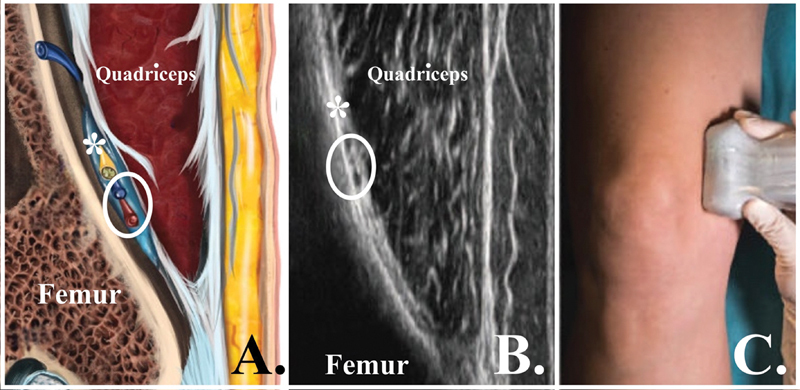
Pontos anatômicos para o bloqueio do nervo genicular superior medial (MSGN). ( **A** ) Imagem esquemática no plano coronal na região metafisária medial distal do fêmur. ( **B** ) Imagem ultrassonográfica obtida com o transdutor no plano coronal na região metafisária medial distal do fêmur. ( **C** ) Transdutor no plano coronal na região metafisária medial distal do fêmur. *Nervo genicular superior medial; círculo branco, veia e artéria genicular superior medial.

Uma solução de 20 mL, composta por 8 mL de ropivacaína (10 mg/mL), 10 mL de dextrose a 50%, mais 2 mL de dexametasona (4 mg/2,5 mL) foi preparada e utilizada para bloqueios do ramo genicular guiados por ultrassom. 11 Então, foram injetados 5 mL cada nos ramos geniculares femoral medial, femoral lateral e tibial medial ( Figs. 3 , 4 e 5 ). Os 5 mL restantes foram misturados aos 15 mL de BMA e infiltrados de maneira intra-articular no joelho em questão ( Fig. 6 ).

**Fig. 5:**
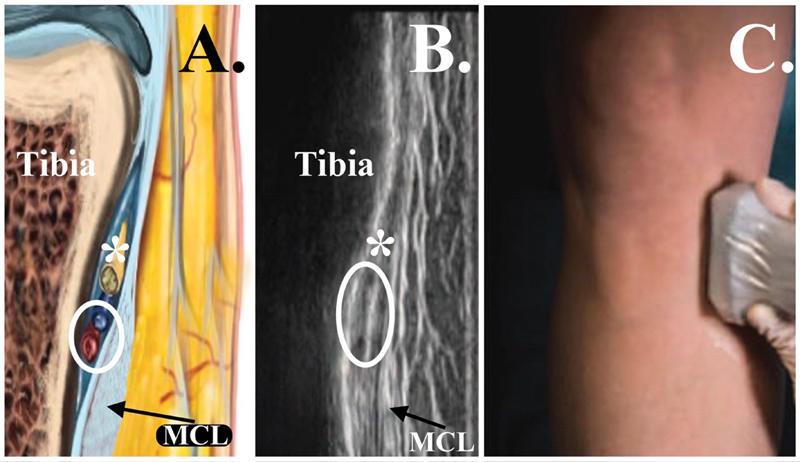
Pontos anatômicos para o bloqueio do nervo genicular inferior medial (MIGN). ( **A** ) Imagem esquemática no plano coronal na região metafisária medial proximal da tíbia. ( **B** ) Imagem ultrassonográfica obtida com o transdutor no plano coronal na região metafisária medial proximal da tíbia. ( **C** ) Transdutor no plano coronal na região metafisária medial proximal da tíbia. *Nervo genicular inferior medial; círculo branco, veia e artéria genicular inferior medial. Abreviação: MCL, ligamento colateral medial.

**Fig. 6:**
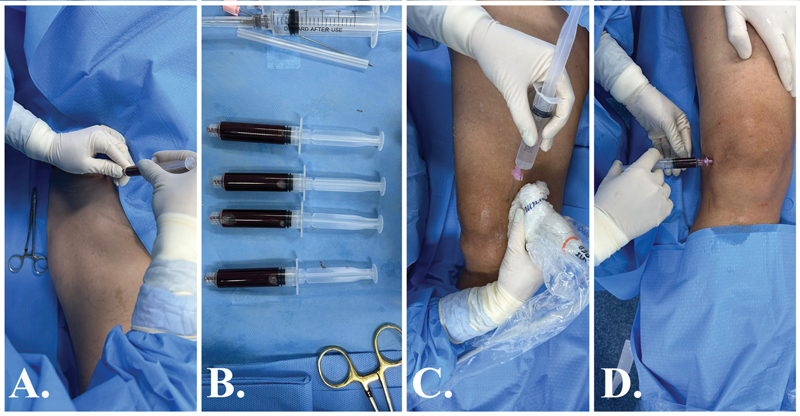
Grupo 1 (Intervenção), tratamento com BMA. ( **A** ) BMA da espinha ilíaca anterossuperior com seringas heparinizadas. ( **B** ) Aproximadamente 15 mL de medula óssea. ( **C** ) Uma solução de 20 mL foi preparada com 8 mL de ropivacaína (10 mg/mL), 10 mL de dextrose 50%, mais 2 mL de dexametasona (4 mg/2,5 mL). Esta solução foi usada para bloqueios do ramo genicular guiados por ultrassom. Desta solução, 5 mL foram aplicados nos ramos geniculares femoral medial, femoral lateral e tibial medial, cada. ( **D** ) Os 5 mL restantes da solução anterior (ropivacaína + dextrose + dexametasona) foram misturados aos 15 mL de BMA e infiltrados intra-articularmente no joelho em questão.

### Grupo 2 (Controle): Infiltração Articular de Corticosteroide e Bloqueio Genicular, Padrões da Instituição

Uma solução de 20 mL, composta por 8 mL de ropivacaína (10 mg/mL), 10 mL de dextrose a 50%, mais 2 mL de dexametasona (4 mg/2,5 mL) foi preparada. Ela foi utilizada para bloqueios do ramo genicular guiados por ultrassom. Então, foram injetados 5 mL cada nos ramos geniculares femoral medial, femoral lateral e tibial medial. Os 5 mL restantes deveriam ser infiltrados intra-articularmente no joelho em questão. Este procedimento é padrão em nossa instituição e a única diferença em comparação ao grupo 1 é que o grupo controle não recebeu a infiltração de BMA.

### Avaliação do Paciente

Todos os pacientes foram encorajados a interromper o uso de AINEs 2 semanas antes e várias semanas após o tratamento. Se os pacientes sentissem dor após o procedimento, a medicação de resgate com opioides era prescrita por até 5 dias. Os pacientes foram aconselhados a evitar atividades que pudessem exacerbar a dor durante todo o protocolo de reabilitação, que começou com repouso e deambulação em casa e/ou na comunidade. A progressão das atividades físicas incluiu natação ou exercícios de baixo impacto, seguidos de caminhada, treinamento de resistência, corrida e, finalmente, avanço para a atividade funcional completa.

As variáveis analisadas em cada grupo foram idade, sexo e lateralidade. A avaliação da resposta terapêutica utilizou o Índice de Osteoartrite das Universidades de Western Ontario e McMaster (WOMAC) validado e padronizado para a língua nativa do paciente. 12 13 Esse questionário contém 17 questões sobre o nível de dificuldade na realização de atividades da vida diária, dor e rigidez para avaliar a funcionalidade do paciente. Quanto maior a pontuação, pior a função. 14 Este questionário usa um critério de diferença clinicamente relevante, uma possível redução de 16% da pontuação total adquirida antes da intervenção. 15 Os pacientes foram avaliados antes do procedimento e 1, 3 e 6 meses após a infiltração. A classificação de Kellgren e Lawrence (KL) foi utilizada na avaliação pré-infiltração. 9

### Metodologia de Análise de Dados

As variáveis categóricas e numéricas foram tabuladas e analisadas usando o software R (R Foundation for Statistical Computing, Viena, Áustria) para Mac OS X, que forneceu medidas de tendência central, valores percentuais e dispersão.

Para avaliação comparativa da eficácia dos tratamentos com BMA e padrões com infiltração articular com corticoides e bloqueio genicular, foram utilizados testes estatísticos não paramétricos devido à distribuição dos dados. O teste U de Mann-Whitney foi aplicado para comparar os escores do WOMAC (dor, rigidez e atividade física) entre os dois grupos em cada um dos quatro momentos de avaliação (pré-intervenção, e aos 1, 3 e 6 meses após o tratamento). Este teste foi escolhido porque não assume uma distribuição normal dos dados e é adequado para amostras independentes.

A comparação das pontuações do WOMAC (dor, rigidez e atividade física) de cada paciente antes do procedimento e após 6 meses utilizou o teste dos postos sinalizados de Wilcoxon, adequado para amostras não paramétricas pareadas.

A investigação das relações entre idade e pontuações do WOMAC utilizou a correlação de Spearman. O teste de qui-quadrado foi aplicado para examinar as relações entre sexo e lateralidade com as pontuações categorizadas como dor intensa ou não, rigidez e atividade física.

As análises foram consideradas estatisticamente significativas com IC de 95% e valor de *p* inferior a 0,05.

## Resultados

Após 6 meses de acompanhamento, o estudo foi concluído com 35 pacientes, 17 do grupo 1 (intervenção com BMA autólogo) e 18 do grupo 2 (controle com infiltração articular de corticoides e bloqueio genicular). A idade média dos participantes foi de aproximadamente 58 anos, com 58,06 no grupo 1 e 57,78 no grupo 2. Em relação à idade, lateralidade e sexo, não foram observadas diferenças estatisticamente significativas entre os grupos 1 e 2. Em termos de distribuição por sexo, o grupo 1 foi composto por 12 mulheres e cinco homens, enquanto o grupo 2 tinha 13 mulheres e cinco homens.

**Fig. 7:**
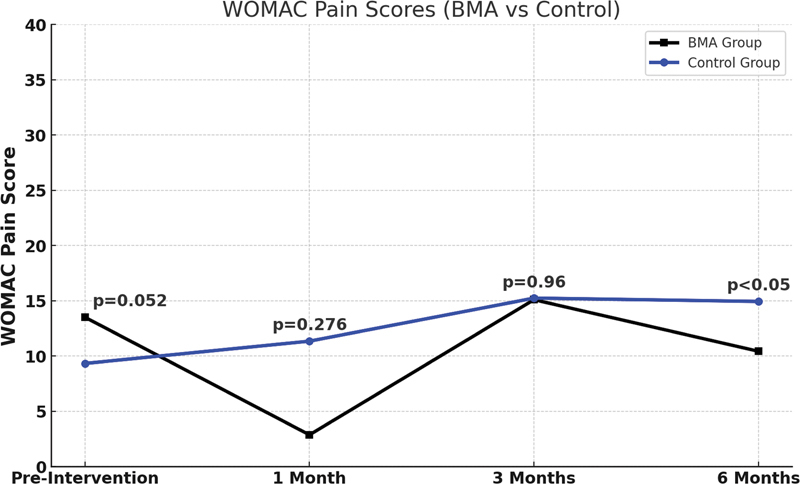
Os escores do WOMAC – Dor (BMA vs. Controle). A pontuação entre os dois grupos em cada um dos quatro momentos de avaliação (pré-intervenção, e em 1, 3 e 6 meses após o tratamento). Essa subseção avalia a intensidade da dor durante várias atividades, como caminhar, subir escadas, deitar-se e ficar em pé. Quanto maior sua pontuação, maior a gravidade dos sintomas e a limitação funcional dos pacientes. O valor de *p* corresponde ao teste *t* independente entre os dois grupos em cada um dos quatro momentos de avaliação.

Em relação ao WOMAC – Dor, a comparação entre os grupos 1 e 2 antes do bloqueio não revelou diferenças estatisticamente significativas ( *p* = 0,052). Nos acompanhamentos de 1 mês e 3 meses, as diferenças também não foram significativas ( *p* = 0,276 e 0,960, respectivamente). Aos 6 meses, houve diferença significativa, com o grupo BMA apresentando menor pontuação de dor em comparação ao grupo controle ( *p* = 0,030), como mostrado na Fig. 7 .

No WOMAC – Rigidez, a comparação dos grupos 1 e 2 não revelou diferenças estatisticamente significativas em nenhum dos períodos avaliados ( *p* = 0,627, 0,789 e 0,097, em 1, 3 e 6 meses, respectivamente).

No WOMAC – Atividade Física, a comparação dos grupos 1 e 2 não revelou diferenças estatisticamente significativas em nenhum dos períodos avaliados ( *p* = 0,894, *p* = 0,960 e *p* = 0,114, em 1, 3 e 6 meses, respectivamente).

**Fig. 8:**
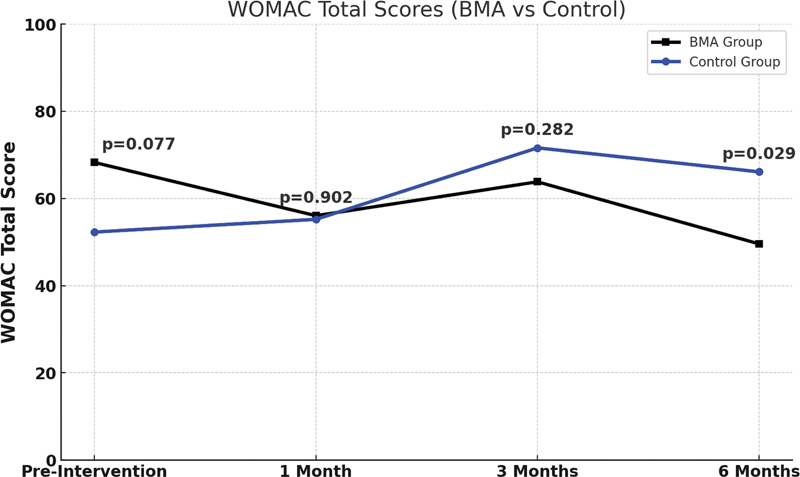
Pontuações totais do WOMAC (BMA vs. Controle). As pontuações totais entre os dois grupos em cada um dos quatro momentos de avaliação (pré-intervenção, e em 1, 3 e 6 meses após o tratamento). A pontuação total é a soma das pontuações das três subescalas (dor, rigidez e função física). Quanto maior a pontuação total, maior a gravidade dos sintomas e a limitação funcional dos pacientes. O valor de *p* corresponde ao teste t independente entre os dois grupos em cada um dos quatro momentos de avaliação.

Entretanto, à comparação do escore total ao longo de 6 meses, o grupo 1 (BMA) apresentou melhores resultados. Porém, embora haja uma tendência à diferença, ela não é estatisticamente significativa em de 5% ( *p* > 0,05), como notado na Fig. 8 .

A correlação entre idade e rigidez aos 6 meses foi moderadamente positiva e estatisticamente significativa (ρ = 0,366, *p* = 0,031), sugerindo que pacientes mais velhos podem apresentar maior rigidez ao longo do tempo, independentemente da técnica utilizada. Não foram encontradas associações significativas entre sexo ou lateralidade e escores WOMAC em nenhum dos períodos avaliados.

Estes resultados indicam que o tratamento com BMA pode oferecer benefícios significativos na redução da dor em pacientes com OA de joelho em comparação ao tratamento padrão de infiltração com corticosteroides articulares e bloqueio genicular. No entanto, apesar de ambos os tratamentos mostrarem melhora na atividade física e nos escores de rigidez ao longo do tempo, não houve diferenças significativas entre os grupos nestas pontuações ( Tabela 1 ).

**Tabela 1:** Os escores WOMAC nos grupos 1 (BMA) e 2 (controle)

**Grupo 1 (BMA)**	**Pré-intervenção**			**1 mês pós-intervenção**		
**Paciente**	**Dor**	**Rigidez**	**Funcionalidade física**	**Dor**	**Rigidez**	**Funcionalidade física**
1	5,2	2,08	16,6	4,1	1,04	51,04
2	11,4	8,03	35	11,4	5,2	45,8
3	17,7	3,12	70,8	3,1	1,04	8,3
4	10,4	2,08	15,6	1,04	0	52,08
5	10,4	2,8	44,7	3,1	0	8,3
6	11,4	8,3	52,2	13,5	3,1	68,7
7	17,2	6,25	36,4	10,4	2,08	51,04
8	9,3	7,2	50	11,4	5,2	45,8
9	14,5	3,1	55,2	11,4	6,2	50
10	9,3	2,08	40,6	2,08	0	30,2
11	17,7	6,2	66,6	13,5	5,2	52,08
12	20,8	8,3	93,7	4,1	6,2	8,3
13	20,8	8,3	68,75	3,1	1,04	51,04
14	17,2	8,08	40,6	4,1	0	12,5
15	11,4	6,25	44,7	13,5	0	8,3
16	10,4	8,3	66,6	27	6,25	58,3
17	14,5	6,25	36,4	13,5	6,2	45,8
**Grupo 1 (BMA)**	**3 meses pós-intervenção**			**6 meses pós-intervenção**		
**Paciente**	**Dor**	**Rigidez**	**Funcionalidade física**	**Dor**	**Rigidez**	**Funcionalidade física**
1	9,3	6,2	15,6	3,1	1,04	8,3
2	17,2	2,08	36,4	11,4	1,04	30,2
3	20,8	8,08	40,6	13,5	3,1	45,8
4	17,2	8,08	66,6	3,1	0	68,7
5	17,7	2,8	44,7	11,4	5,2	58,3
6	11,4	8,3	52,2	4,1	1,04	30,2
7	17,2	6,25	36,4	13,5	5,2	45,8
8	9,3	7,2	50	27	5,2	51,4
9	14,5	3,1	55,2	13,5	1,04	52,05
10	9,3	2,08	40,6	3,1	0	8,3
11	17,7	6,2	66,6	27	3,1	45,8
12	20,8	8,3	93,7	11,4	6,2	8,3
13	20,8	8,3	68,75	3,1	6,2	51,04
14	17,2	8,08	40,6	4,1	0	12,5
15	11,4	6,25	44,7	13,5	1,4	8,3
16	10,4	8,3	66,6	11,4	6,25	58,3
17	14,5	6,25	36,4	3,1	6,2	30,2
**Grupo 2 (controle)**	**Pré-intervenção**			**1 mês pós-intervenção**		
**Paciente**	**Dor**	**Rigidez**	**Funcionalidade física**	**Dor**	**Rigidez**	**Funcionalidade física**
1	4,1	1,04	51,04	9,3	0	52,08
2	11,4	5,2	45,8	10,4	3,1	51,04
3	3,1	1,04	8,3	11,4	2,08	30,2
4	1,04	0	52,08	3,1	5,2	45,8
5	3,1	0	8,3	4,1	1,04	30,8
6	13,5	3,1	68,7	13,5	6,2	68,7
7	10,4	2,08	51,04	10,4	2,08	52,08
8	11,4	5,2	45,8	13,5	2,08	45,8
9	11,4	6,2	50	11,4	6,2	30,2
10	2,08	0	30,2	27	1,04	45,8
11	13,5	5,2	52,08	13,5	5,2	52,08
12	4,1	6,2	8,3	4,1	6,2	8,3
13	3,1	1,04	51,04	3,1	1,04	51,04
14	4,1	0	12,5	4,1	0	12,5
15	13,5	0	8,3	13,5	0	30,2
16	27	6,25	58,3	27	6,25	51,04
17	13,5	6,2	45,8	13,5	6,2	30,2
18	17,7	8,3	68,75	11,4	2,08	45,8
**Grupo 2 (controle)**	**3 Meses Pós-intervenção**			**6 Meses Pós-intervenção**		
**Paciente**	**Dor**	**Rigidez**	**Funcionalidade física**	**Dor**	**Rigidez**	**Funcionalidade física**
1	13,5	6,2	15,6	20,8	6,25	45,8
2	17,2	2,08	36,4	4,1	3,1	30,2
3	20,8	8,08	40,6	13,5	3,1	52,05
4	17,2	8,08	66,6	20,8	5,2	68,7
5	17,7	2,8	44,7	11,4	5,2	58,3
6	11,4	8,3	52,2	4,1	6,25	51,4
7	17,2	6,25	36,4	13,5	5,2	45,8
8	9,3	7,2	50	27	5,2	51,4
9	14,5	3,1	55,2	13,5	1,04	52,05
10	9,3	2,08	40,6	13,5	3,1	30,2
11	17,7	6,2	66,6	27	5,2	45,8
12	20,8	8,3	93,7	11,4	6,2	68,75
13	20,8	8,3	68,75	17,7	6,2	51,04
14	17,2	8,08	40,6	13,5	1,4	12,5
15	11,4	6,25	44,7	13,5	3,1	30,2
16	10,4	8,3	66,6	11,4	5,2	58,3
17	14,5	6,25	36,4	20,8	6,2	30,2
18	13,5	1,04	52,2	11,4	3,1	58,3

**Abreviações:** BMA, aspirado de medula óssea; WOMAC, índice de osteoartrite das universidades de Western Ontario e McMaster.

## Discussão

Este estudo avaliou a eficácia do tratamento da OA de joelho usando BMA autólogo em comparação à infiltração articular de corticosteroides e bloqueio genicular. Independentemente do grupo, todos os pacientes apresentaram melhora clínica. No entanto, os resultados após 6 meses de acompanhamento indicam uma redução significativa da dor para o grupo tratado com BMA, sugerindo uma melhora na qualidade de vida desses pacientes.

A técnica BMA tem se destacado em várias doenças ortopédicas devido ao seu potencial regenerativo e baixo risco. Especificamente, o BMA fornece um rico suprimento de células regenerativas capazes de diferenciação em vários tipos de tecido, representando uma abordagem promissora no tratamento da OA. Com base na compreensão atual dos mecanismos inflamatórios na OA, o BMA é considerado uma alternativa terapêutica relevante. 5 7 Este tratamento é baseado em MSCs, que são pluripotentes, ou seja, têm a capacidade de se diferenciar em diversos tecidos, incluindo osteócitos, condrócitos, adipócitos, mastócitos, fibroblastos e células precursoras hematopoiéticas. 16 Além disso, essas células têm propriedades imunomoduladoras e podem suprimir a apoptose dos condrócitos. 17

Uma das vantagens do BMA como fonte de MSCs é sua técnica relativamente simples. É um procedimento percutâneo com baixa morbidade por ser uma fonte autóloga e não necessitar de processamento, diferentemente do concentrado de aspirado de medula óssea (BMAC) e das fontes adiposas, que necessitam de várias etapas de processamento. 5 18 Não há um protocolo universalmente padronizado para o processamento. Diferentes clínicas e pesquisadores podem usar parâmetros ligeiramente diferentes. A eficácia do BMAC pode depender da qualidade e pureza da preparação. Quanto a regulamentações e diretrizes, países ou regiões podem ter dispositivos específicos acerca de seu processamento e uso. Em alguns países, a utilização de medula óssea processada só é permitida em protocolos de pesquisa.

O sítio preferencial para obtenção do aspirado geralmente é a crista ilíaca posterior, um local seguro que apresenta menos complicações e possui maior quantidade de MSCs em comparação à crista ilíaca anterior. 19 Entretanto, ainda não há consenso sobre alguns aspectos da técnica, como o posicionamento do paciente, a anestesia e a escolha das agulhas de coleta. 20 21 O principal fator da técnica é a manutenção da pressão de aspiração constante e baixa, optando-se por seringas de 5 ou 10 mL. 22 Isso ocorre porque as MSCs são diluídas no sangue quando aspiradas, fazendo com que 85% das células disponíveis sejam coletadas nos primeiros 2 mL do aspirado. Portanto, a cada 2 mL coletados, a agulha deve avançar 0,5 a 1 cm para otimizar a coleta. 5

Bastos et al. 23 estudaram a eficácia e a segurança de injeções intra-articulares de MSCs estromais autólogas expandidas obtidas por aspiração de medula óssea (± 80 a 100 mL) de ambas as cristas ilíacas posteriores em pacientes com OA de joelho. Esses autores concluíram que injeções intra-articulares de MSCs expandidas sozinhas ou combinadas ao PRP são seguras e têm efeitos benéficos sobre os sintomas em pacientes com OA sintomática do joelho. Esses pacientes foram avaliados com um questionário de funcionalidade e qualidade de vida chamado Knee and Osteoarthritis Outcome Score (KOOS) em intervalos de 1, 2, 3, 6, 9 e 12 meses. Semelhante ao outro estudo, um pico inicial de melhora foi observado nos primeiros 2 meses, com estabilização até o 9° mês e, então, uma ligeira melhora no 12° mês.

Garay-Mendoza et al. 24 conduziram um estudo clínico prospectivo, aberto, de fase I/II para avaliação da segurança e eficácia de uma única injeção intra-articular de células-tronco da medula óssea estimuladas autologamente (BM-SC) em pacientes com OA de joelho. As BM-SC foram obtidas por aspiração e administradas em uma única injeção intra-articular. O grupo controle recebeu apenas paracetamol oral. As pontuações da escala visual analógica (EVA) e do WOMAC foram obtidas 1 semana, 1 mês e 6 meses após o procedimento em ambos os grupos. Os pacientes apresentaram melhora significativa, especialmente na EVA, que foi superior ao grupo controle. Este estudo demonstrou a viabilidade e eficácia de um procedimento que pode ser realizado em regime ambulatorial para tratamento da OA de joelho.

Apesar dos resultados promissores, a eficácia em longo prazo e a otimização dos protocolos de tratamento com BMA ainda requerem validação por mais pesquisas. O potencial regenerativo das MSCs, embora promissor, ainda não é totalmente compreendido. Estudos de porte maior e mais rigorosos são necessários para consolidar o BMA como uma opção terapêutica viável e eficaz para a OA de joelho. 7 Em nosso estudo, a ausência de diferenças significativas entre os grupos em termos de idade e sexo sugere que ele pode ser aplicável a uma ampla gama de pacientes sem a necessidade de seleção com base nesses critérios demográficos. Isso é encorajador, pois indica que os benefícios do tratamento podem ser generalizados para ambos os sexos e uma ampla faixa etária.

Neste contexto, à medida que a medicina regenerativa avança, o uso de BMA para OA de joelho está em uma fase emocionante de desenvolvimento. Uma melhor compreensão de como essas terapias podem ser integradas aos planos de tratamento existentes pode alterar significativamente a abordagem ao gerenciamento da OA, oferecendo novas esperanças para reduzir a dor e melhorar a qualidade de vida dos pacientes.

Em relação às limitações deste estudo, destacamos o tamanho da amostra e a duração do acompanhamento. Infelizmente, tivemos uma perda de acompanhamento de pacientes. Começamos o estudo com 50 pacientes, 25 em cada grupo, e terminamos com apenas 35. Estudos futuros devem abordar essas limitações, idealmente com amostras e períodos de acompanhamento maiores, para confirmar e expandir os achados relatados. Outra limitação do estudo foi que não incluímos dados demográficos importantes, como índice de massa corporal e o grau de OA de cada indivíduo.

## Conclusão

O tratamento com BMA pode reduzir significativamente a dor, podendo levar à melhora da funcionalidade do joelho, sugerindo seu potencial como uma opção terapêutica viável para o tratamento da OA de joelho.
